# Supplementary figures and images for: Enamel Matrix Derivative Decreases Pyroptosis-Related Genes in Macrophages
Source: Int J Mol Sci. 2022 May 3;23(9):5078. doi: 10.3390/ijms23095078 (PMC9099857; doi:10.3390/ijms23095078)

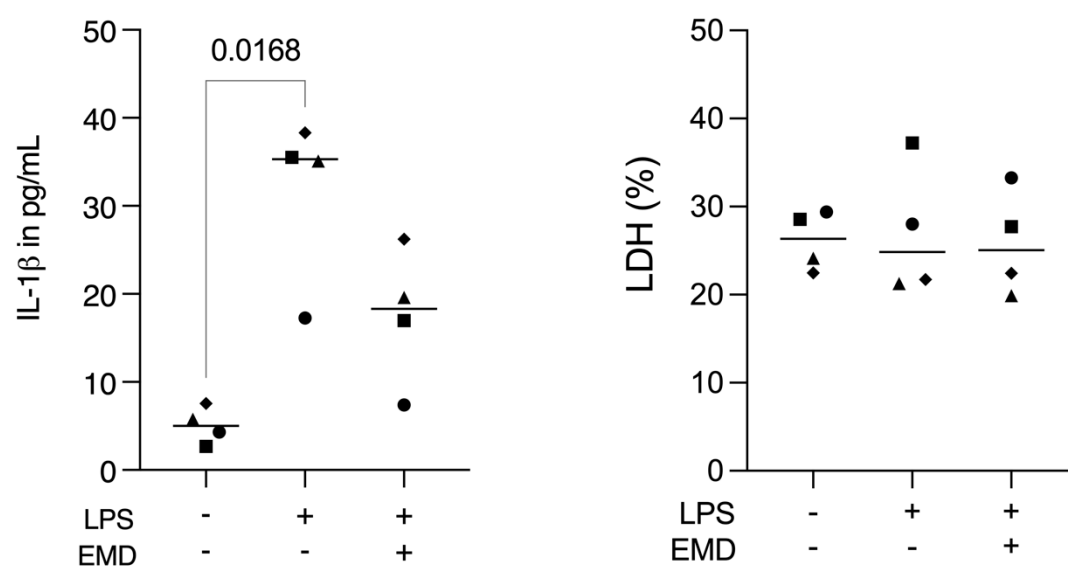

Supplement Figure S1

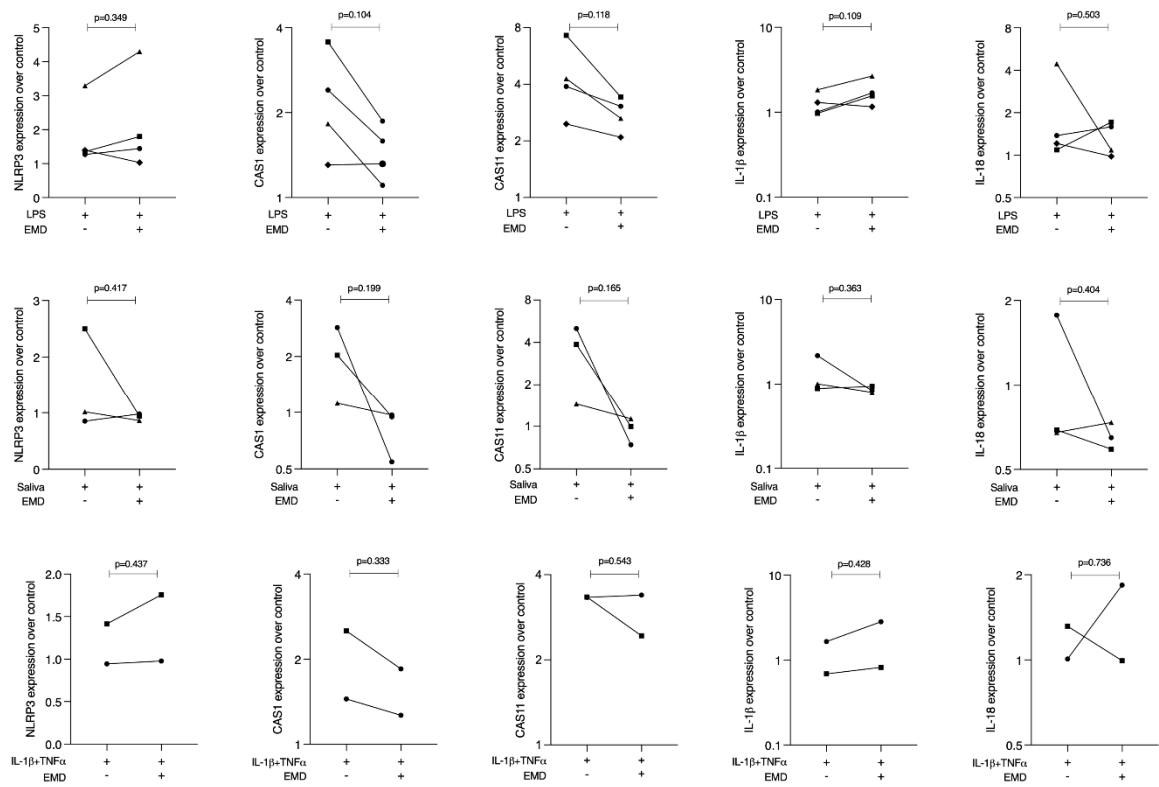

Supplement Figure S2

Supplement: Supplementary file 1 [file ijms-23-05078-s001.zip › ijms-1703507-supplementary.pdf]
